# Chronic Ingestion of Advanced Glycation End Products Induces Degenerative Spinal Changes and Hypertrophy in Aging Pre-Diabetic Mice

**DOI:** 10.1371/journal.pone.0116625

**Published:** 2015-02-10

**Authors:** Svenja Illien-Jünger, Young Lu, Sheeraz A. Qureshi, Andrew C. Hecht, Weijing Cai, Helen Vlassara, Gary E. Striker, James C. Iatridis

**Affiliations:** 1 Leni & Peter W. May Department of Orthopaedics/Icahn School of Medicine at Mount Sinai, New York, NY, United States of America; 2 Department of Geriatrics and Palliative Care, Division of Experimental Diabetes and Aging/Icahn School of Medicine at Mount Sinai, New York, NY, United States of America; 3 Department of Geriatrics and Palliative Care, Division of Experimental Diabetes and Aging, and Division of Nephrology, Department of Medicine/Icahn School of Medicine at Mount Sinai, New York, NY, United States of America; University of Insubria, ITALY

## Abstract

Intervertebral disc (IVD) degeneration and pathological spinal changes are major causes of back pain, which is the top cause of global disability. Obese and diabetic individuals are at increased risk for back pain and musculoskeletal complications. Modern diets contain high levels of advanced glycation end products (AGEs), cyto-toxic components which are known contributors to obesity, diabetes and accelerated aging pathologies. There is little information about potential effects of AGE rich diet on spinal pathology, which may be a contributing cause for back pain which is common in obese and diabetic individuals. This study investigated the role of specific AGE precursors (e.g. methylglyoxal-derivatives (MG)) on IVD and vertebral pathologies in aging C57BL6 mice that were fed isocaloric diets with standard (dMG+) or reduced amounts of MG derivatives (dMG-; containing 60-70% less dMG). dMG+ mice exhibited a pre-diabetic phenotype, as they were insulin resistant but not hyperglycemic. Vertebrae of dMG+ mice displayed increased cortical-thickness and cortical-area, greater MG-AGE accumulation and ectopic calcification in vertebral endplates. IVD morphology of dMG+ mice exhibited ectopic calcification, hypertrophic differentiation and glycosaminoglycan loss relative to dMG- mice. Overall, chronic exposure to dietary AGEs promoted age-accelerated IVD degeneration and vertebral alterations involving ectopic calcification which occurred in parallel with insulin resistance, and which were prevented with dMG- diet. This study described a new mouse model for diet-induced spinal degeneration, and results were in support of the hypothesis that chronic AGE ingestion could be a factor contributing to a pre-diabetic state, ectopic calcifications in spinal tissues, and musculoskeletal complications that are more generally known to occur with chronic diabetic conditions.

## Introduction

The etiology of low back pain is multifactorial and often correlated with intervertebral disc (IVD) degeneration. Genetics, diminished physical activity, overweight and obesity conditions are all strong risk factor for IVD degeneration [1, 2, 3]. Overweight and obesity conditions can be caused by consumption of processed food that are high in advanced glycation endproducts (AGEs). IVD degeneration involves chronic inflammation, which is known to be present in diabetes and other metabolic disorders [4]. In IVD degeneration, chronic inflammation is associated with a catabolic shift of IVD metabolism, increased cell death, and a loss in glycosaminoglycan (GAG) content [5]. These tissue alterations lead to reduced hydration, increased IVD stiffness and loss of overall IVD height [6]. Determining an association of diet, diabetes and spinal pathology, whether causative or correlative, is important in treating complications associated with metabolic disorders and may also shed light on mechanisms for pathological IVD degeneration.

Non-insulin dependent type 2 diabetes mellitus (T2DM) is a worldwide epidemic that affects approximately 25.8 million Americans or 8.3% of the United States population [7]. Alarmingly, about half the United States population over the age of 60 is considered pre-diabetic and therefore at risk of developing clinical diabetes. T2DM affects multiple systems, e.g. the cardiovascular, neurologic, and renal systems. The effect of diabetes and AGEs on musculoskeletal disorders is highly under-explored [8, 9, 10, 11], and the focus of the present work is on AGE induced IVD degeneration.

Emerging evidence suggests that accumulation of reactive glycation reaction intermediates (collectively termed advanced glycation endproducts or AGEs) may be important drivers of IVD cell and tissue level changes that are commonly associated with IVD degeneration [12, 13, 14]. Excess accumulation of AGEs is known to lead to stiffness, brittleness and overall alterations in tissue biomechanics of collagen rich tissues [13, 15, 16, 17]. T2DM is thought to be a predisposing risk factor for the development of spinal pathology such as lumbar disc herniation and spinal stenosis [18, 19]. Furthermore, T2DM is associated with changes in bone quality [20] leading to increased bone fragility and brittleness and therefore is considered to be a risk factor for vertebral or other bone fractures [21, 22, 23].

AGEs are formed from the non-enzymatic reaction or Maillard reactions between reducing sugars and free amino groups on proteins, lipids and nucleic acids [24]. Common AGEs, including N^ε^-carboxymethyl-lysine (CML), pentosidine, and glucosepane are associated with protein structural changes, while reactive AGE precursors, such as the cytotoxic metabolite methylglyoxal (MG) and its derivatives such as methylglyoxal-hydroimidazolone-1 (MG-H1) are linked to cellular injury [15, 25, 26, 27, 28].

Endogenous AGE formation occurs slowly in normal aging, which in part is driven by sugars, and in diabetes hyperglycemia can accelerate the accumulation of AGEs [29]. In addition, the western style diet contains a substantial proportion of industrially processed foods that have been shown to contain high levels of AGEs [24]. Uribarri et al. reported that healthy individuals on a regular diet ingest approximately 18’000 kU AGEs/day, which is contributing to serum levels of ~14.0 U/ml CML and ~1.1 nmol/ml MG. With 23’000 kU AGEs/day and serum levels of ~24.2 U/ml CML and ~3.5 nmol/ml MG these values are increased in diabetic individuals. On low AGE diet (~50% less AGEs/day) serum CML and MG levels were significantly reduced by ~25% in healthy and ~30% in diabetic individuals [30], indicating that AGEs derived from diet are significant contributors to AGE serum levels.

Ten percent of orally consumed AGEs are absorbed through the gut and of that 7% interact with tissues and cells. Under high AGE ingestion, the small but continuous influx of AGEs is shown to contribute to the development of vascular/renal age- and diabetes associated complications [24, 31, 32]. In contrast, restriction of oral AGEs prevents these conditions [33]; thus, the diet is an important source of AGEs in addition or prior to diabetes. Among the consequences of chronic AGE consumption can be osteoarthritis [34, 35] and atherosclerotic calcification [36], which underlie the chronic complications of diabetes [29, 37].

The IVD is reportedly the musculoskeletal connective tissue most strongly affected by aging in diabetic sand rats [10]. We recently showed that diabetes accelerated degenerative changes to vertebrae and IVDs in a streptozotocin-induced diabetic mice [38], and these degenerative changes were found associated with the accumulation of AGEs in both the IVD and the vertebral bodies. Administration of anti-AGE and anti-inflammatory medications reduced the diabetes induced spinal degeneration, further suggesting that AGEs play an important role in spinal degeneration [38]. However, there is no causal study to evaluate if chronic AGE ingestion in diets can accelerate degenerative changes to the spine.

The purpose of this study is to evaluate the effects of oral AGEs separately from AGEs that derive from hyperglycemia, by evaluating the effects of exogenously derived well characterized AGE precursors on the spinal structures of non-diabetic mice. The data suggests that chronic oral AGE ingestion can have significant adverse effects on IVDs separately from, or prior to, hyperglycemia and that their restriction can effectively prevent these effects pointing to the need of further research and new therapeutic strategies.

## Research Design and Methods

### Research Design

After weaning, C57BL/6 mice were assigned for life to two pair-fed groups receiving either a low AGE chow (n = 12), produced without the use of heat (dMG^-^, containing MG: 0.7×10^4^ nmol/day; and CML: 14×10^4^ U/day; Test Diet Low AGE 5053; WF Fisher & Son CO, NJ, USA), or a low AGE chow supplemented with synthetic MG-BSA (n = 9) [39]. The dMG^-^ and dMG^+^ diets were identical in caloric and nutritional content. The dMG^+^ diet contained MG-BSA (2.0×10^4^ nmol/day) and CML (22×10^4^ U/day), at levels which were equivalent to those of standard mouse chow and contained nearly twice as much AGEs than MG^-^ diet (Table 1).

**Table 1:** Characteristics of dMG^+^ and dMG^-^ mice at sacrifice.

	**dMG^+^**	**dMG^-^**	**p**
Body Weight (g)	33.3 ± 3.8	28.7 ±2.8	*
White body fat (g)	2.36 ±1.0	0.89 ±0.26	**
Food intake (g/day)	4.9 ± 1.7	4.8 ± 2.8	
Food MG intake (nmol/day)	1.9 ±0.7×10^4^	0.7 ±0.4×10^4^	*
Food CML intake (U/day)	24.4 ±8.5×10^4^	16.3 ±9.5×10^4^	*
Serum MG (nmol/ml)	1.84 ± 0.58	0.83 ± 0.14	**
Serum CML (U/ml)	43.9 ± 10.3	26.0 ±6.4	**
Fasting blood glucose (mg/dl)	81.8 ± 13.8	82.6 ± 9.7	
Fasting insulin (nmol/l)	0.41 ± 0.24	0.24 ± 0.07	*
Adiponectin (ug/ml)	7.8 ± 3.5	13.7 ± 4.2	**
Leptin (ng/ml)	22.7 ± 3.1	10.3 ± 3.1	**
8-Isoprostane (pg/ml)	267 ± 112.1	88.0 ± 19	**

All mice were 18 month old wild type C57BL6; [dMG^+^] denotes mice on a MG-supplemented low-AGE diet; [dMG^−^] denotes mice on non-thermally-treated low-AGE diet. Data are means ± SD; *p < 0.05; **p < 0.01 between dMG^+^ and dMG^-^ mice.

Body weights, total food intake, food MG intake, and food CML intake were noted and blood was taken for measurements of serum MG, serum CML, fasting blood glucose, fasting insulin, Adiponectin, Leptin, and 8-Isoprostane. White adipose tissue was carefully dissected from abdominal space and weighed. Mice were sacrificed at 18 months of age. This study was carried out in accordance with the recommendations in the Guide for the Care and Use of Laboratory Animals of the National Institutes of Health. (Department of Health, Education, and Welfare, NIH 78–23, 1996) and all animal protocols were approved by the Mount Sinai Institutional Animal Care and Use Committee (protocol # 02–0480–00002–01-PD). All euthanasia was performed using carbon dioxide inhalation and all efforts were made to minimize suffering.

### AGE Determination

Two common AGE markers (CML and MG-H1) in serum and chow were determined by competitive ELISAs, as previously described [25].

### Spine harvest and µCt

Following sacrifice, lumbar spines were dissected and fixed in 10% buffered formalin phosphate (Fisher Scientific, Fair Lawn, NJ, USA). Spines were washed in PBS and µCt analyses of trabecular and cortical bone of vertebrae and end plates were performed using a Pre-Clinical Specimen Micro-computed Tomography system (eXplore Locus SP; GE Healthcare, London, Ontario, Canada). Lumbar vertebrae (L4) were used to measure bone structure as described below. Three-dimensional images of the entire lumbar spine were obtained with a short scan at an 8.7 micron voxel size (acquisition parameters: 400 views at 0.5 degree increment (9 pictures/view) 80kVp, 80uA, 3 second exposure) and cross-sections were analyzed for the amount of trabecular bone (trabecular number, trabecular spaces, trabecular thickness, and bone volume, fraction) and cortical bone (cortical thickness, cortical area, total area, and cortical area fraction). Contours were selected by manually approximating the contour outline with the polygon advanced ROI tool, then the “Shrink Wrap” tool (Microview ABA 2.2; GE healthcare) was used to generate a finer outline of the contour with a resolution of 10 nodes/10 pixels. Intervertebral disc (L4-L5) and lumbar vertebrae (L4) height measurements were calculated by specifying contour coordinates in Microview ABA 2.2 (GE healthcare) and calculating the difference between the coordinates via a custom script in MATLAB 2010 (Mathworks). Vertebrae and IVD heights were calculated with the software by subtracting the y coordinates of nodes which are aligned on the x coordinate. The disc height index (DHI) was calculated based on mid coronal IVD and vertebrae height measurements (IVD height/L4 height method adapted from Masuda et al. 2005 [40]). The DHI reflects the IVD height relative to the vertebrae to account for inter animal size variations.

### Endplate cell quantification

Images of the entire superior and inferior endplates of lumbar vertebrae (L3–5) were captured at 20x magnification. Regions of interest were defined manually (ImageJ; http://rsbweb.nih.gov/ij/) and cells (chondrocytes and osteocytes) within the region of interest were counted by two blinded observers using ImageJ. The values were averaged and the number of cells/mm^2^ was calculated.

### Histology and immunohistochemistry

Calcified IVD-vertebrae segments of L3–4 and L4–5 were embedded in methacrylate (dMG^+^ n = 10; dMG^-^ n = 11) and 5 µm thick sagittal sections were used for histology and immunohistochemistry. All sections were de-plasticized in in a series of toluene, petroleum-ether and Ethylene Glycol Mono Ethyl Ether. GAG content was visualized by extended FAST stain [41] (abbreviation: F = Fast-green, A = Alcian blue, S = Safranin-O, T = Tartrazine) and von Kossa [42] staining was performed to detect calcification. Immunohistochemistry was performed for Collagen 10 alpha-1 (COL-X), MG, CML, tumor necrosis factor α (TNFα), and a disintegrin and metalloproteinase with thrombospondin motifs-5 (ADAMTS-5). De-plasticized sections were washed in distilled water, incubated for 5 minutes in Proteinase K (s3020, DAKO) followed by another wash. Unspecific bindings were blocked with blocking solution (DAKO) and incubated with the primary antibody over night at 4°C. The following day, sections were rinsed with distilled water and incubated for 30 minutes in secondary anti body. Samples were rinsed again and incubated for 1 minute in a chromogenic staining solution (diaminobenzidine, DAKO), washed in distilled water and counterstained with toluidine blue for 30 seconds, rinsed in distilled water, Ethylene Glycol Mono Ethyl Ether, and toluene and then cover-slipped. Negative controls were treated the same but were incubated with a universal negative control (DAKO) instead of the primary antibody.

### Statistical analyses

For statistical analyses unpaired t-tests were used (GraphPad Prism5) and a p-value < 0.05 was considered significant. Error Bars were displayed as ±SD.

## Results

### General observations of the MG-fed mouse groups

dMG^+^ mice at 18 months had elevated fasting plasma insulin but were not hyperglycemic, compared to dMG^-^ mice (table 1). They also had significantly higher serum MG (p < 0.01), serum CML (p < 0.01), fasting insulin (p < 0.0001) adiponectin (p < 0.01), leptin (p < 0.01), 8-Isoprostane (p < 0.01), white body fat (p < 0.0002) and body weight (p < 0.05; table 1) indicating that dMG^+^ mice became insulin resistance, a complication that has been observed previously in aged mice on dMG^+^ diet or standard diet which is also high in AGEs [43].

### Chronic dMG affected bone structure

**Figure 1:**
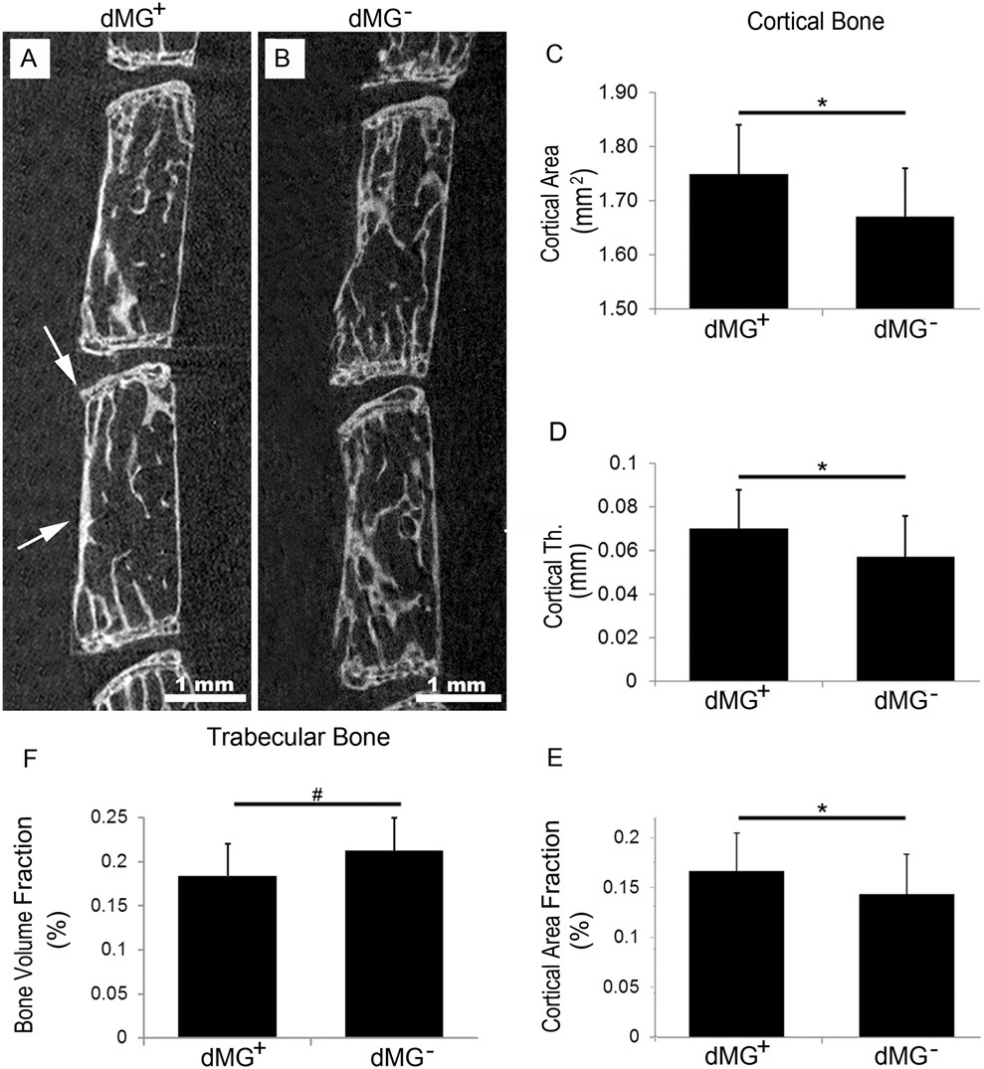
dMG intake induces pathologic changes in vertebrae and EP. Representative µCT images demonstrate ectopic calcification in vertebrae and EP of (A) dMG^+^ and (B) dMG^-^ mice. White arrows demonstrate calcification of the superior EP and cortical bone in dMG^+^ mice. Scale bar = 1 mm. µCT analyses demonstrate increased (C) cortical area, (D) cortical thickness and (E) cortical area fraction but decreased (F) bone volume fraction of dMG^+^ vertebrae, *p < 0.05, #p < 0.09.

In vertebrae, chronic dMG intake lead to significantly increased vertebral cortical thickness (*p* = 0.0299) and area (*p* = 0.0477) while the total cross-sectional area did not change (*p* = 0.4337). This difference in cortical bone morphology was further supported by the cortical area fraction, which was increased after chronic dMG intake (trend: *p* = 0.0631; Table 2, Fig. 1). Concurrently, in the trabecular bone the bone volume fraction (the ratio of the segmented bone volume to the total volume of interest) tended to be decreased after dMG intake (*p* = 0.0878), however no differences in trabecular thickness, number, separation, or bone mineral density, were observed. Noteworthy, the average of each parameter was decreased in dMG^+^ mice compared to dMG^+^ mice (Table 2).

**Table 2:** µCT analyses.

			**dMG^+^**	**dMG^-^**	**p-value**
**vertebrae**	**Trabecular**	**BV/TV (%)**	**0.184 ±0.037**	**0.213 ± 0.037**	***p* = 0.088**
		Tb.Th. (mm)	0.021 ±0.002	0.022 ± 0.002	*p* = 0.256
		Tb.N. (1/mm)	8.84 ±2.07	9.60 ± 2.12	*p* = 0.317
		Tb.Sp. (mm)	0.098 ±0.029	0.084 ± 0.03	*p* = 0.153
	**cortical**	***Cr.Th. (mm)***	***0.070 ± 0.018***	***0.057 ± 0.019***	***p = 0.029***
		Cr.Ar. (mm^2^)	0.294 ±0.077	0.237 ± 0.081	*p* = 0.434
		***Tt.Ar. (mm*** *^2^*)	***1.75 ±0.09***	***1.67 ± 0.09***	***p = 0.047***
		**Cr.Ar./Tt.Ar. (%)**	**0.167 ±0.04**	**0.143 ± 0.04**	***p* = 0.063**
**IVD**		***Coronal DHI***	***0.075 ±0.008***	***0.083 ± 0.007***	***p = 0.014***
		***Coronal IVD height (mm)***	***0.269 ±0.025***	***0.298 ± 0.028***	***p = 0.022***

Abbreviations: bone volume fraction (BV/TV), trabecular thickness (Tb.Th.), trabecular number (Tb.N.), trabecular spacing (Tb.Sp.), cortical thickness (Cr.Th.), cortical area (Cr. Ar.), total area (Tt.Ar.), cortical area fraction (Cr.Ar./Tt.Ar.), disc height index (DHI). Data are means ± SD. ***Bold & italic = significance*** p < 0.05 **bold = trend** p < 0.09.

### Chronic dMG is associated with decreased IVD height

Mid-coronal disc height index and IVD height were both significantly decreased in dMG^+^ mice compared to dMG^-^ mice (Table 2). No differences were observed for sagittal disc height measurements (data not shown).

### Ectopic calcification deposits detected in dMG^+^ mice

**Figure 2:**
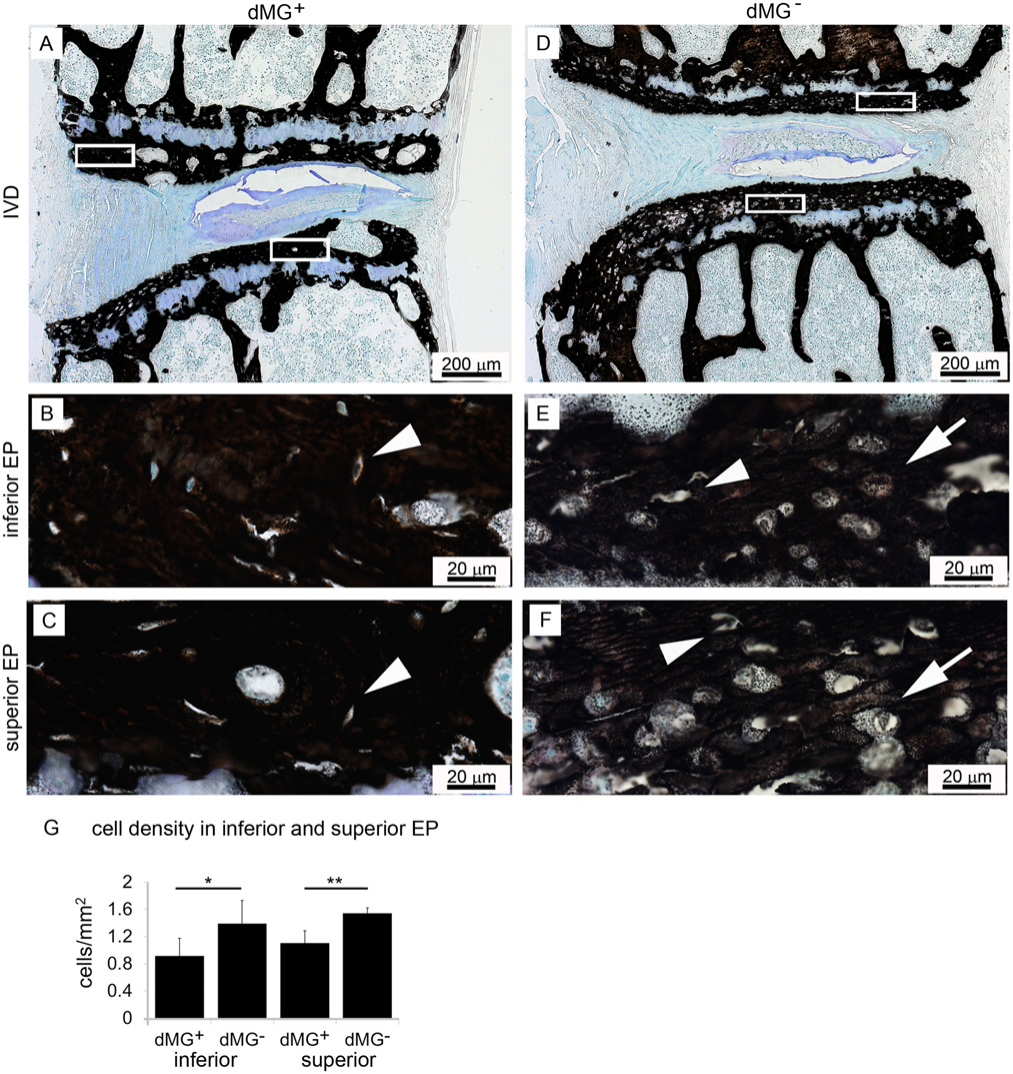
EPs of dMG+ exhibit a compact structure with increased calcification and cell loss. Representative images of Von Kossa staining (dark-brownish color) for (A-C) dMG^+^ and (D-F) dMG^-^ mice. GAG and nuclei are visualized with Toluidine blue counterstain. (A-C) Dark brownish von Kossa staining in EPs of dMG^+^ mice reveals compact EPs indicating increased calcification in the (B) inferior and (C) superior EPs which contain few, mainly osteocyte like cells (arrow heads). EPs dMG^-^ mice contain osteocyte like (arrow heads) and chondrocyte like (arrows) cells. (G) Significantly fewer cells populate the inferior and superior EPs of dMG+ mice compared to dMG^-^ mice; *p < 0.05, **p = 0.002.

Increased calcification of EP and IVD in dMG^+^ mice was observed by von Kossa stain (Fig. 2). EPs of dMG^+^ mice appeared compact and dark, an indication for calcified tissues (Fig. 2 B+D). Further, EPs of dMG^+^ mice contained significantly fewer cells (superior EP: dMG^+^: 0.915±0.258 cell/mm^2^; dMG^-^: 1.3.89±0.342 cell/mm^2;^ *p* = 0.002; inferior EP: dMG^+^: 1.105±0.180 cell/mm^2^; dMG^-^: 1.540±0.083 cell/mm^2^, *p* = 0.049) compared to dMG^-^ EPs. The cell population between groups appeared also different: dMG^+^ EPs contained mainly small osteoclast (Fig. 2 B+C, arrow head) while the EPs of dMG^-^ mice contained both, small osteoclast (arrow head) and larger chondrocytes (arrow, Fig. 2 E+F).

### High COL-X staining was mainly observed in IVD cells from dMG^+^ mice

**Figure 3:**
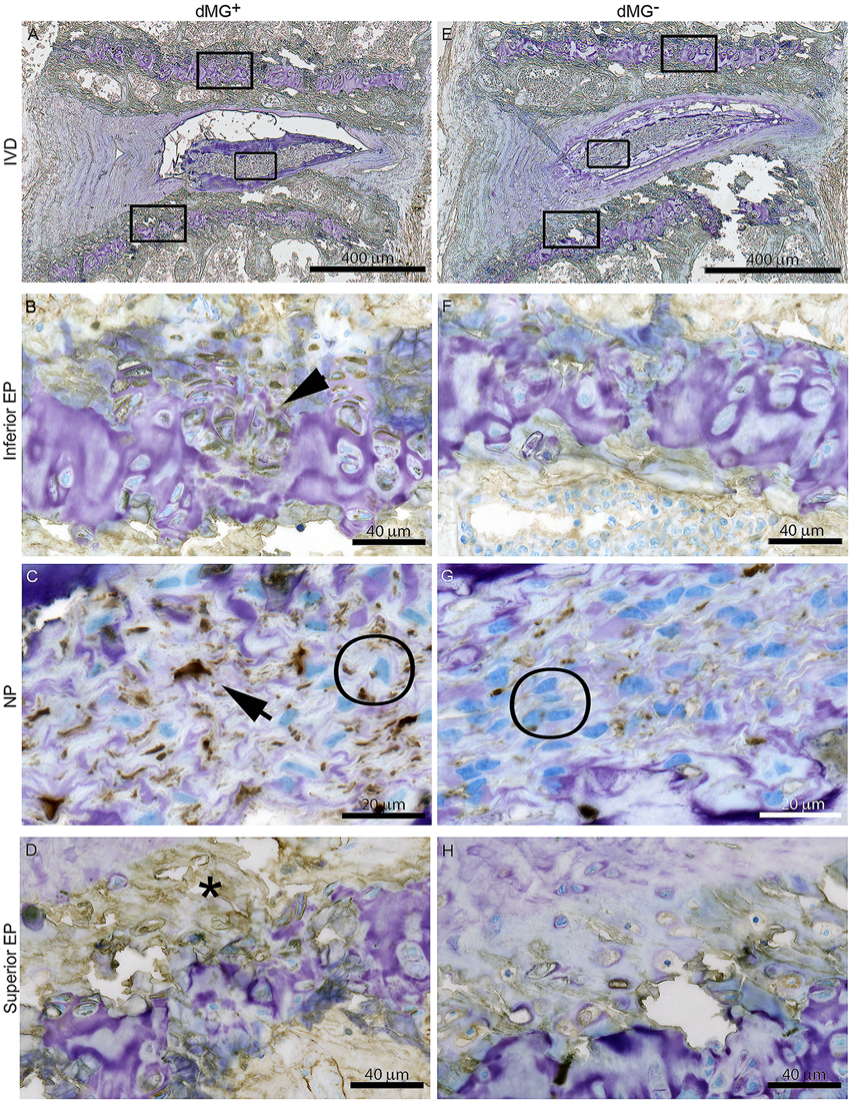
Hypertrophy is mainly detected in NP and EPs of dMG^+^ mice. Representative images of COL-X immunohistochemistry for (A-D) dMG^+^ and (E-H) dMG^-^ mice. Brown stain indicates COL-X presence, GAG and nuclei are visualized with toluidine blue counterstain; (C+G) Compared to dMG^-^ the NP cells of dMG^+^ mice appeared bigger and edged shaped, with cytoplasm positive COL-X (circle) which is also highly expressed in the extracellular matrix (arrow). Increased COL-X abundance is also observed in the (B) Inferior and (D) Superior EPs of dMG^+^ mice (asterisk). COL-X positive areas surrounding cells within the cartilaginous (arrow head). (G) Some COL-X stain is present in the extracellular matrix of the more organized notochordal NP of dMG^-^ mice. (F+H) minor COL-X staining is observed in (F) inferior and (H) superior EPs of dMG^-^ mice.

The EP, extracellular matrix and NP cells of dMG^+^ mice were highly positively for COL-X; The NP cells also appeared bigger than the NP cells of dMG^-^ mice (Fig. 3), indicating hypertrophic differentiation of these cells. In dMG^+^ mice the Notochordal like cells appeared more disorganized (Fig. 3C), which is a sign of degeneration in mature mice [38, 44]. In dMG^-^ mice the notochordal like cells of the NP were organized and appeared like a notochordal band which was surrounded by the mature NP tissue, and more representative of normal aging.

### Low GAG content within the NP of dMG^+^ mice

**Figure 4:**
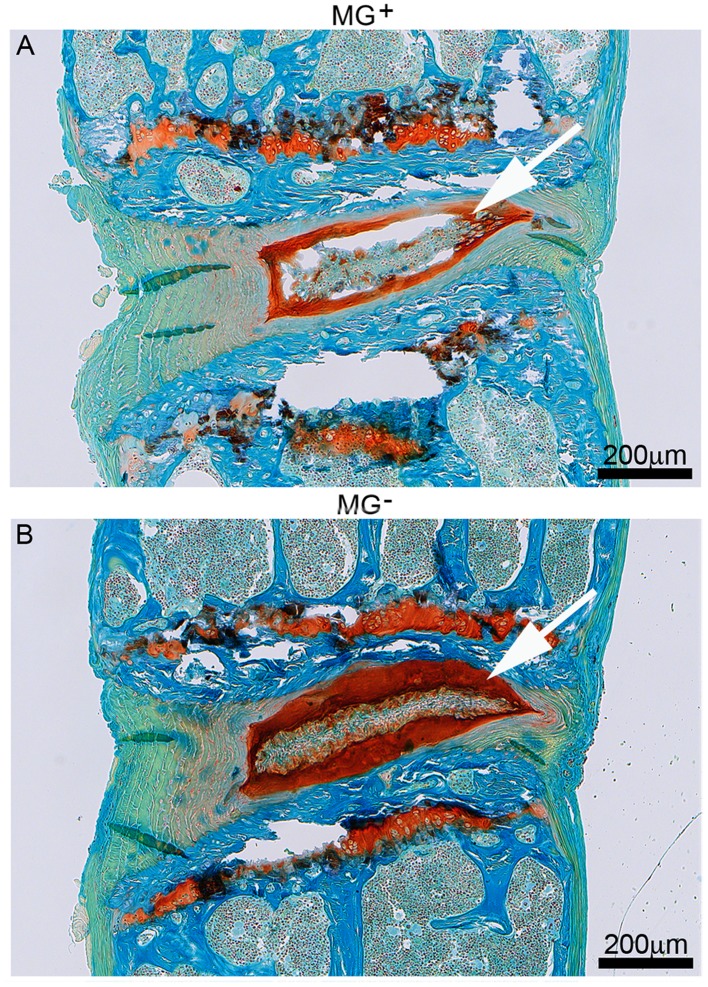
Chronic MG-H1 accumulation causes GAG loss. Representative extended FAST stain in (A) dMG^+^ and (B) dMG^-^ mice. (A) White arrow marks extensive GAG (red) loss of the NP and ruptures indicate potential cracking or artifact in the IVD tissue that we speculate to be associated with ectopic calcifications in the NP region. (B) The notochordal band of dMG^-^ mice appears dense and is embedded in NP extracellular matrix that is rich in GAG (white arrow).

Extended FAST stain demonstrated a weakly stained NP region in the dMG^+^ mice as compared to the GAG rich region in dMG^-^ mice which was thick and intensely stained (Fig. 4).

### Increased MG-derivatives in vertebrae and EPs of MG^+^ mice

**Figure 5:**
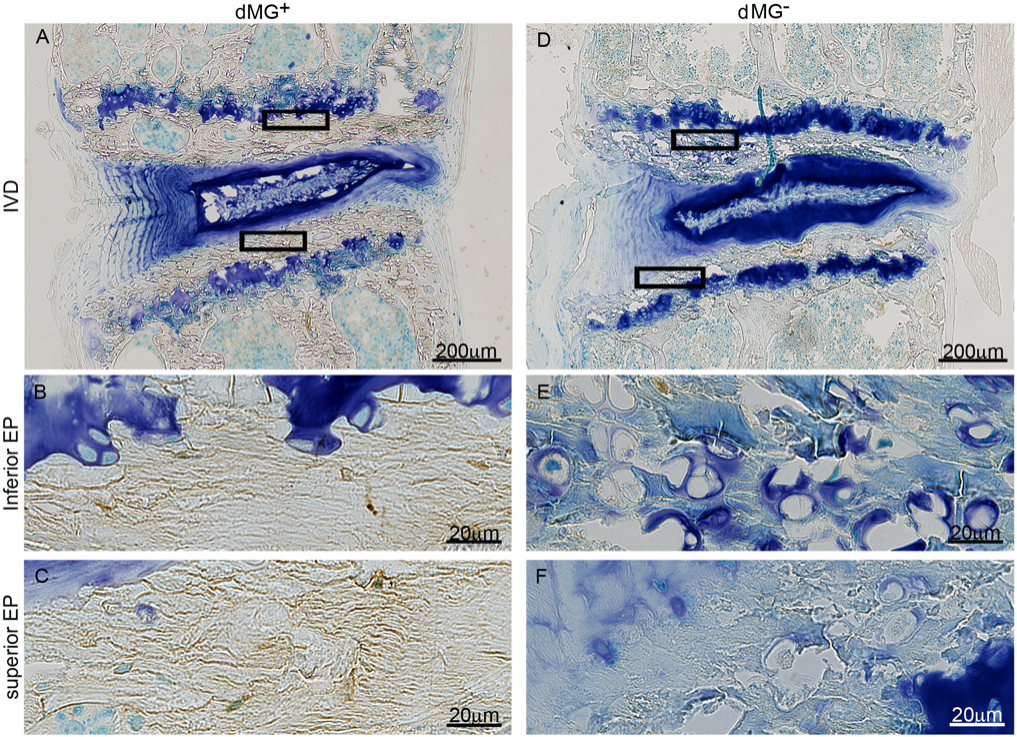
MG-H1 accumulates in vertebral structures and the NP of dMG^+^ mice. Representative images of MG-H1 immunohistochemistry for (A-C) dMG^+^ and (D-F) dMG^-^ mice (A+B); Brown stain indicates MG-H1 presence, GAG and nuclei are visualized with toluidine blue counterstain; MG-H1 is highly expressed in the EP of (A-C) dMG^+^ mice compared to (D-F) dMG^-^ mice. Boxes mark 40x magnification of (B+E) inferior and (C+F) superior EPs.

Immunostaining revealed MG-H1-like epitopes in vertebrae and EP of dMG^+^ mice. However, the NP regions of these mice did not stain positively, suggesting MG-H1-like derivatives accumulated predominantly in the endplates and not within the IVD region of these animals. Only weak MG-H1 staining was detected in vertebrae or EP of dMG^-^ mice (Fig. 5).

## Discussion

**Figure 6:**
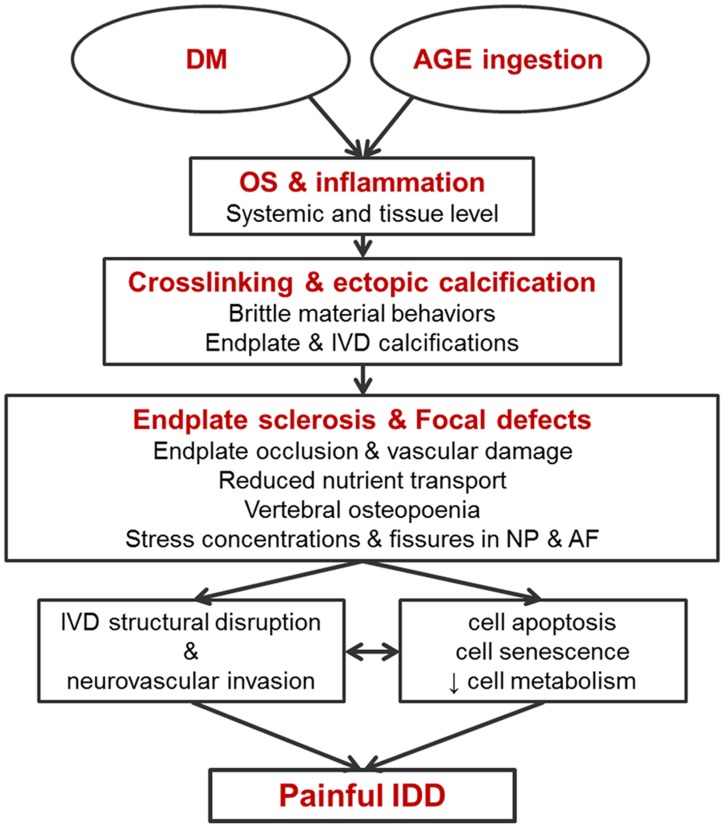
Hypothetical model for dMG induced IVD degeneration showing the potential of dMG, independent of hyperglycemia being responsible for AGE to promote IVD degeneration, independently of hyperglycemia.

This study investigated how diet that that is high in AGEs alters spinal structures. We here tested the hypothesis that chronic ingestion of AGEs, cyto-toxic compounds prevalent in animal and human diet [45, 46], can negatively influence spinal structures as a function of age, separate from hyperglycemia. Chronic exposure to MG-derivatives led to increased levels of circulating AGEs (i.e., increased serum levels for CML and MG), and age-accelerated degenerative changes in the spine relative to the dMG^-^ mice. The early pathological changes observed in the spine included increased cortical thickening in the vertebrae, calcification of endplates, decreased IVD height and GAG content, and increased expression of COL-X, suggesting hypertrophic differentiation of NP cells,. The presented data indicate that high levels of AGEs (and certain AGE precursors) in food, independently of food quantity is an important factor in maintaining spinal health and may play a role in accelerated aging of spinal structures. Together, results suggest a novel hypothetical model that high amounts of MG-derivatives resulted in ectopic calcifications that could accelerate degeneration by impeding nutritional pathways and creating focal defects (Fig. 6), although this hypothesis requires further testing.

The dMG^+^ mice exhibited increased vertebral cortical thickness, cortical area, and cortical area fraction while the trabecular bone volume fraction was slightly decreased. The bone volume fraction reflects the contribution of the structure to the mechanical properties of the trabecular bone [47], and suggests that the cortical bone had to compensate for the decreased bone volume fraction, even though no differences between Trabecular number, thickness or spacing were detected. Studies have demonstrated that T2DM patients had reduced bone quality in the vertebrae [48], tibia and femur [49] compared to their age matched controls [20, 37], and patients with type 1 and T2DM are known to have increased risk of fractures [20]. *In vivo* studies on partially insulin-deficient rats, demonstrated that diabetes induced deleterious changes on long-bone micro-architecture [50]. Further, T2DM negatively affected femur and vertebrae in Zucker Diabetic Fatty rats [51].

AGEs induce pathologic calcification in the vasculature [52, 53, 54], and it is likely that AGE accumulation promoted the increased calcification and decreased cell density observed in vertebral EPs of dMG^+^ mice compared to dMG^-^ mice. These changes indicate that AGE accumulation rather than simple aging led to these pathological changes and therefore might be a potential mechanism for accelerated IVD degeneration in dMG^+^ mice. The compact vertebral EPs in dMG^+^ mice could have caused the diminished nutrient and oxygen content, and elevated accumulation of metabolic by-products such as lactic acid which could have led to a drop in IVD cell viability, loss of extracellular matrix production and increased catabolism as has been previously described for the IVD [55]. In this hypothetically dysfunctional nutritional state, IVD cells demonstrated by up-regulation of COL-X, a known marker for hypertrophy which has been shown to be increased in degenerated IVDs [56, 57, 58].

Calcified EPs of dMG^+^ mice contained significantly fewer cells compared to dMG^-^ mice, and a reduction of chondrocytes in EPs is a sign for IVD degeneration [59]. Further, adequate numbers of osteocyte lacunae are essential for healthy bone remodeling as well as maintenance of overall biomechanical quality of the bone [60]. Bone lacunae contain osteocytes which are believed to initiate activation of osteoblasts and/or osteoclasts, and mediate exchange of nutrient and waste products between the Haversian canals and osteocyte network, thereby playing a vital role in regulating bone mineral homeostasis [61, 62]. The observed decrease in cell density within EPs with their compact and dense appearance of dMG^+^ mice EPs suggests poorer bone quality [63] as bone is susceptible to AGE accumulation, and non-enzymatic glycation increased its propensity to fracture [64].

Loss of cells and occlusion of lacunae through calcification of the subchondral bone likely limited the diffusion through the EP which is the main route for nutrient supply into the large avascular human IVD [65]. Such nutritional compromise in the IVD would result in decreased glucose concentration and accumulation of metabolic waste products would increase within the IVD [66], leading to degenerative changes in the IVD.

The presence of MG-derivatives in the diet had detrimental effects on the structure on the NP of dMG^+^ mice. Notochordal cells appeared disorganized, and resembled a phenotype consistent with early degenerative changes [38]. COL-X accumulation surrounding the notochordal cells suggested the presence of calcified deposits which might have led to their edged shaped morphology (Fig. 3). Pathological calcification could be a factor leading to the observed degenerative patterns in the NP of dMG^+^ mice which included GAG loss, thinned GAG rich-region, disorganized notochordal band, and focal defects (Fig. 4). Calcification in the IVD has been associated with IVD degeneration in an ovine model where calcified IVDs contained less GAG due to hydroxyapatite depositions [67]. Crystal depositions in degenerated IVDs are relatively common and might be partly causative for extracellular matrix disruption and activation of matrix metalloproteinases and contribution to degenerative changes in IVDs [68], and the current study provides insights into potential causes for these ectopic calcifications. GAG maintenance is a crucial factor enabling the IVD to retain water and to resist compression during loading and in diabetic patients the GAG synthesis rate has been shown to be decreased [69]. The dMG^-^ mice had a healthy IVD with NP rich in GAG, suggesting that low dietary MG ingestion could have a protective effect against IVD aging.

The important role of EP involvement in AGE mediated IVD degeneration was demonstrated by MG-H1 immunohistostaining (Fig. 5). Only minor MG-H1 accumulation was observed in spinal structures of dMG^-^ mice, while dMG^+^ mice stained strongly positive for MG-H1 in vertebrae and endplates. Interestingly, dMG^-^ and dMG^+^ mice demonstrated only minor MG-H1 staining within the NP or annulus fibrosus regions of the IVD, suggesting that the observed degenerative changes within the IVD were likely not due to the direct effects of AGEs on NP tissue. We believe the degenerative changes observed in the IVDs were associated with calcifications and MG-H1 accumulation in the vertebrae and endplates.

A limitation of this study is that AGE-derivatives were only quantified in serum. In the spine, we provide qualitative immunohistochemical analyses that demonstrated abundant MG-positive deposits in vertebral bones and EPs indicating the presence of AGEs in spinal structures and their potential relevance in the observed changes. It is challenging to obtain sufficient tissue to measure small amounts of protein that accumulate in mouse IVDs, and this study prioritized immunohistochemical analyses to provide presence and localization information over quantitative protein analyses.

Our results indicate that food processing methods that increase AGE content can lead to accelerated aging of spinal structures. All mice were fed an isocaloric diet but compared to dMG^-^ mice; dMG^+^ mice had greater body weight, white fat content, abdominal obesity, and were insulin resistant but not hyperglycemic (table 1). It cannot be excluded that the observed changes within the spinal structures of dMG^+^ mice were due to the increased body weight or other metabolic effects rather than the effects of excessive AGE accumulation, although the weight gain of dMG^+^ mice was relatively modest and seems to be an unlikely cause. For example, obesity was identified as a risk factor for osteoarthritis in non-weight bearing regions like the hand [70]. Furthermore, Griffin et al. demonstrated that increased joint loading in obese mice which were fed a very high fat diet was not sufficient to explain the increased incidence of knee osteoarthritis [71].

The dMG^+^ intake was associated with elevated fasting plasma insulin and leptin and suppressed adiponectin levels in dMG^+^, indicating that AGEs can alter spinal structures independently of excess intake of nutrients. AGEs impair the insulin signaling pathway, and previous studies demonstrated that food-derived MG-derivatives can contribute to insulin resistance in aged mice in quantities like they are found in standard diet [43].

In the present study dMG also led to a pre-diabetic phenotype, which was indicated by the development of insulin resistance in dMG^+^ mice and we cannot exclude insulin as a potential cause for the observed degenerative changes in vertebral structures of dMG^+^ mice [72]. It is possible that spinal health is also dependent on the health of other organ systems and therefore further studies would be needed to differentiate between localized and systemic effects of AGEs on the IVD. Dietary MG restriction therefore might protect spinal structures in two ways: first by direct prevention of calcification and occlusion of the EPs and second by preserving insulin sensitivity.

To our knowledge, this is the first study to show how a diet supplemented with MG-derivatives can contribute to musculoskeletal diseases. Results indicated that chronic ingestion of MG-derivatives can accelerate spinal degeneration via the proposed hypothetical model including ectopic calcification and hypertrophy in endplates, and accelerated vertebral and IVD degeneration. This fundamental information on AGE accumulation and diet induced spinal pathology may help to develop strategies to improve spinal health, mobility, and quality of life in diabetic and pre-diabetic patients.

There are certain practical ways to reduce the amount of AGEs from exogenous sources, e.g. by changing the diet: A meal rich in complex carbohydrates, fresh fruits and vegetables with moderate intake of meat will significantly lower daily AGEs intake (detailed information about AGE content in food and it’s implication on general health are published elsewhere) [46, 73]. Furthermore, the observed pathophysiology of AGE accumulation and ectopic calcifications provides a novel mechanism that may explain some of the observed degenerative changes commonly reported in the spine with aging and may eventually lead to early and minimally invasive interventions to treat painful musculoskeletal conditions.
